# The GluN3-containing NMDA receptors

**DOI:** 10.1080/19336950.2025.2490308

**Published:** 2025-04-16

**Authors:** Kunlong Xiong, Shulei Lou, Zuoyu Lian, Yunlin Wu, Zengwei Kou

**Affiliations:** a Department of Pulmonary and Critical Care Medicine, Affiliated First Hospital of Ningbo University, Ningbo, Zhejiang, China; b Institute of Hospital Management, Linyi People’s Hospital, Linyi, Shandong, China; c Department of General Practice, Cicheng Town Central Health Center, Ningbo, Zhejiang, China; d Hospital Infection Control Section, Affiliated First Hospital of Ningbo University, Ningbo, Zhejiang, China; e Department of Laboratory Medicine and Pathobiology, Temerty Faculty of Medicine, University of Toronto, Toronto, ON, Canada

**Keywords:** Ionotropic glutamate receptors, ligand-gated ion channels, synaptic transmission, pathology and physiology, protein prediction

## Abstract

N-methyl-D-aspartate receptors (NMDARs) are heterotetrameric ion channels that play crucial roles in brain function. Among all the NMDAR subtypes, GluN1-N3 receptors exhibit unique agonist binding and gating properties. Unlike “conventional” GluN1-N2 receptors, which require both glycine and glutamate for activation, GluN1-N3 receptors are activated solely by glycine. Furthermore, GluN1-N3 receptors display faster desensitization, reduced Ca^2+^ permeability, and lower sensitivity to Mg^2+^ blockage compared to GluN1-N2 receptors. Due to these characteristics, GluN1-N3 receptors are thought to play critical roles in eliminating redundant synapses and pruning spines in early stages of brain development. Recent studies have advanced pharmacological tools for specifically targeting GluN1-N3 receptors and provided direct evidence of these glycine-activated excitatory receptors in native brain tissue. The structural basis of GluN1-N3 receptors has also been elucidated through cryo-EM and artificial intelligence. These findings highlight that GluN1-N3 receptors are not only involved in essential brain functions but also present potential targets for drug development.

## Introduction

N-methyl-D-aspartate receptors (NMDARs), a subfamily of ionotropic glutamate receptors (iGluRs), serve as critical “coincidence detectors,” requiring both membrane depolarization to relieve Mg^2+^ block and agonist binding for activation [1]. These receptors play essential roles in synaptic signaling at glutamatergic synapses [2] and are vital for brain processes such as synaptic plasticity, learning, and memory [3]. Alterations in NMDAR expression or dysfunction are implicated in various neurological and psychiatric disorders [4,5].

NMDARs are composed of seven subunits, including the obligatory GluN1 subunit, which binds glycine, and the alternative GluN2 subunits (GluN2A-GluN2D), which bind glutamate. Additionally, the alternative GluN3 subunits (GluN3A and GluN3B) also bind glycine. Functional NMDARs are heterotetrameric ligand-activated ion channels, assembled from two GluN1 subunits and two identical (di-heterotetrameric, di-) or different (tri-heterotetrameric, tri-) alternative subunits. Conventional di-GluN1-N2 NMDARs are composed of two GluN1 subunits and two GluN2 subunits (GluN2A-D), requiring both glycine and glutamate for activation. These receptors form nonselective cation channels that are preferentially permeable to Na^+^ and Ca^2 +^, while also permeable to K^+^ [6]. However, the net K^+^ flux is relatively small near resting membrane potentials, which are close to its reversal potential. The GluN3 subunits can assemble with GluN1 to form unconventional GluN1-N3 di-heteromeric channels, which are activated by glycine alone [7]. Additionally, GluN3 subunits are incorporated into tri-heteromeric receptors, such as GluN1-N2-N3 and GluN1-N3-N3, as shown through immunoprecipitation, immunostaining, and electrophysiology studies [8–12].

GluN3-containing receptors differ significantly from conventional GluN1-N2 receptors. These differences make the GluN3-containing receptors complementary to GluN1-N2 in both physiological and pathological contexts, making them promising targets for fine-tuning brain function and developing therapeutic interventions for brain diseases. In this article, we review GluN3-containing receptors in terms of development, biophysical properties, physiological functions, and structural properties, aiming to provide insights for future studies while summarizing recent findings.

## Basic information of GluN3 subunits

**Figure 1.**
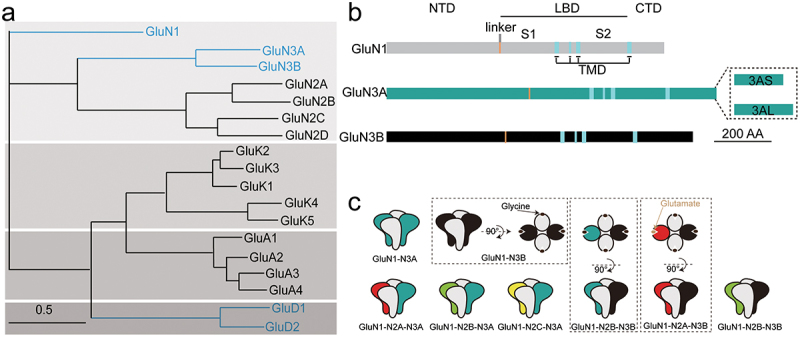
Subunit homology of iGluRs, sequence alignment of GluN1 and GluN3, schematic model of di- or tri-heteromeric GluN3-containing NMDARs. a. The relationship between the iGluRs subunits. The iGluRs are classified into the NMDARs, AMPARs (α-amino-3-hydroxy-5-methyl-4-isoxazole propionic acid receptors), KARs (Kainate receptors), and GluDRs. The sequences Q05586, Q12879.1, Q13224.3, Q14957.3, Q15399.2, Q9R1M7.1, O60391.2, P42261.2, P42262.3, P42263.2, P48058.2, P39086.1, Q13002.1, Q13003.3, Q16099.2, Q16478.2, Q9ULK0.2, O43424.2, were analyzed. b. Schematic structure of the GluN1, GluN3A, and GluN3B subunits. c. Schematic models of several di- and tri-heterotetrameric GluN3-containing NMDARs with “1-3-1-3” or “1-2-1-3” stoichiometries.

GluN3A (formerly called χ-1 subunit)and GluN3B have been cloned from brain tissue in 1995 [13] and 2001 [14]. The gene encoding GluN3A contains 10 exons, encodes 1115 amino acids, and shares 57% protein sequence identity with GluN3B, which is 901 amino acid long and is encoded by 9 exons. From an evolutionary point of view, the GluN3 family is closer to GluN1, since GluN3A and GluN3B share 21% and 48% sequence identity to GluN1. The sequence identity of GluN3A subunit to GluN2A is 25%, GluN2B is 24%, GluN2C is 24%, and GluN2D is 27% [13]. For GluN3B, the sequence identity to GluN2A, N2B, N2C, and N2D are 20%, 20%, 25%, and 30% [15]. While the sequence identity of GluN3A to non-NMDA iGluRs is 22% on average (Figure 1(a)), indicate GluN3 is a unique NMDARs subunit lineage in evolutionary. The topology of NMDARs can be divided into four domains: the extracellular N-terminal domain (NTD), the ligand-binding domain (LBD), the transmembrane domain (TMD), and the intracellular C-terminal domain (CTD) (Figure 1(b)). Two types of GluN3A exist in the brain due to alternative splicing in the CTD. The GluN3A-long isoform is 20-amino acids longer than the GluN3A-short isoform. An intriguing phenomenon is observed in GluN3B: the “RERLR” region in GluN1, which functions as an endoplasmic reticulum (ER) retention signal, does not prevent the membrane expression of GluN1-GluN3B. Instead, the membrane expression of GluN3B is regulated by a sequence located at its C-terminal region (residues 952–985) [16]. As alternative subunits, GluN3-containing NMDARs can exist in several combinatorial modes, including di-heteromeric GluN1-N3, tri-heteromeric GluN1-N3-N3, and tri-heteromeric GluN1-N2-N3 (Figure 1(c)).

## Spatial-temporal and subcellular distribution of GluN3 subunit

The GluN1 subunit is widely expressed throughout the brain during all developmental stages, whereas other NMDAR subunits exhibit distinct spatial and temporal expression patterns [17]. The GluN3 subunits follow specific spatiotemporal expression profiles, which vary across developmental stages, brain regions, and cell types. GluN3A is highly expressed during early brain development but decreases in adulthood, while GluN3B is prominent during adolescence and maintains a considerable expression level in adults [11,12,18].

### Temporal and regional distribution of GluN3 subunits

**Figure 2.**
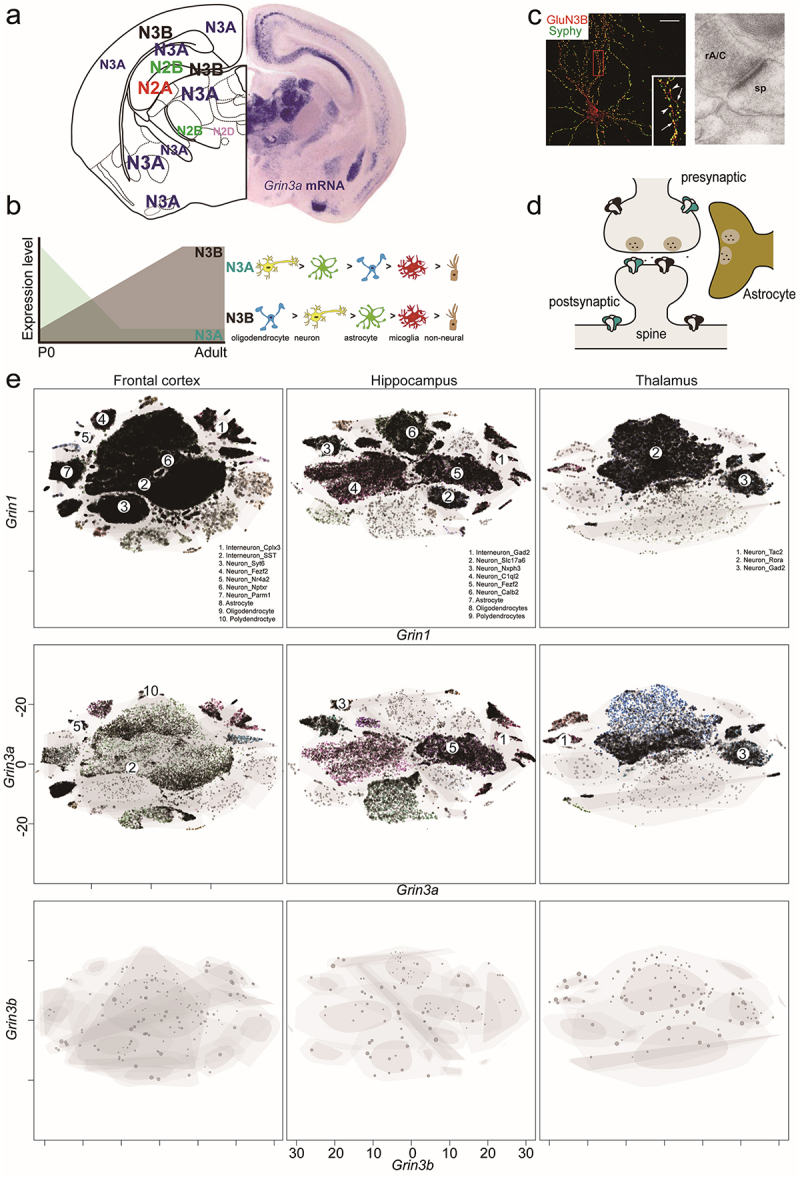
The spatial and subcellular expression pattern of GluN3. a. A schematic diagram illustrates GluN3 and other NMDAR subunits expression pattern in the adult brain. b. Schematic representation of changes in GluN3A and GluN3B expression during brain development and comparison of expression in major neuronal cells. c. GluN3B is expressed in the cultured neurons and co-located with the synaptophysin. Electron micrograph demonstrating GluN3B localized in the dendritic spine. rA/C, recurrent associational-commissural terminal; sp, dendritic spine. d. GluN1-GluN3 can be in both the presynaptic and postsynaptic areas. e. t-distributed stochastic neighbor embedding (t-sne) plots for the frontal cortex, the hippocampus, and the thalamus global expression patterns of *Grin1*, *Grin3a*, and *Grin3b*. Excerpt from [20] (a), [23, 28] (c), [25] (e), and these figures have been reproduced with permission from Oxford University Press (a), Springer nature (c), Elsevier (c), and Frontiers (e), respectively.

The GluN3A subunit is highly expressed in the neonatal brain, particularly in the cortex and hippocampus, reaching peak expression around postnatal day 8 (P8) before declining [11]. In adults, it remains highly expressed in restricted regions, such as the medial habenula (MHb) [19]. However, a recent study by Murillo et al. demonstrated that GluN3A is expressed in multiple brain regions across P8, P14, and adulthood (Figure 2(a,b)), suggesting its involvement in adult brain functions [20], which will be discussed in Section 4.

The GluN3B subunit exhibits a different expression pattern. While traditionally thought to be restricted to the brainstem and spinal cord [7,14,18,21], some studies suggest that it may be expressed more widely across the brain during development, akin to GluN1 [22]. Its expression level increases postnatally, particularly between P8 and P14, coinciding with the sharp decline of GluN3A [23].

### Cell-type-specific expression of GluN3A and Glun3B

Single-cell transcriptomic studies have provided a more detailed view of GluN3 subunit expression across different cell types. GluN3A is detected in both neurons and glial cells, with the highest expression levels in oligodendrocytes, followed by neurons, astrocytes, and microglia [24] (Figure 2(a,b)). In the cortex, GluN3A is enriched in SST (somatostatin)-positive inhibitory interneurons and GAD (glutamate decarboxylase)-positive neurons [24], consistent with its negative modulatory role in synaptic function [19]. In contrast, GluN3B shows a distinct expression pattern. In the frontal cortex, it is predominantly found in GAD-positive inhibitory neurons, like GluN3A [24]. In the hippocampus, GluN3B is highly expressed in GAD2-positive neurons, Fezf2 (forebrain embryonic zinc finger 2)-positive neurons, and oligodendrocytes, whereas GluN3A is less abundant. In the thalamus, GluN3A is primarily found in Tac2 (Tachykinin 2)-positive and GAD2-positive neurons, while GluN3B expression is nearly undetectable (Figure 2(e)) [25]. GluN3B is predominantly detected in polydendrocytes, neurons, astrocytes, and microglia, with its highest expression in polydendrocytes (Figure 2(b)).

### Subcellular localization of GluN3 subunits

At the subcellular level, GluN3A and GluN3B show distinct localization patterns: GluN3A colocalizes with both the somatodendritic domain marker (MAP2, microtubule-associated protein 2) and the axonal marker (SMI-32), indicating a broad cellular distribution. While NMDARs are traditionally enriched in the postsynaptic domain, GluN3A has been found in pre-, post-, and peri-synaptic domains. Immunogold electron microscopy confirmed GluN3A localization in both pre- and postsynaptic regions [26,27] (Figure 2(c,d)). GluN3B, on the other hand, is primarily found in the somatodendritic domain but is rarely detected in axons, suggesting a preference for postsynaptic localization [26,27]. GluN3B colocalizes strongly with synaptophysin/PSD (postsynaptic density) puncta, supporting its enrichment in postsynaptic sites [23]. At the synapse level, GluN3B is detected in both pre- and postsynaptic regions in the mossy fiber terminal cells [28] (Figure 2(c,d)).

## Structural basis of GluN3-containing NMDARs

There is a growing body of cryo-EM (cryogenic electron microscopy) and crystal structures of full-length conventional GluN1-N2 NMDARs, which provide basic information for intra- and inter-receptor comparison at a near-atomic level. Conventional NMDARs can form a bouquet-like heterotetrameric complex in a “1,2,1,2” conformation with two obligatory GluN1, occupying the “A,” “C” positions, and two identical or different alternative GluN2 subunits, located at “B,” “D” positions [29–31]. The NTD of GluN3A can be oxidatively crosslinked by exchanging arginine 319 with a cysteine residue(R319C), which implies the GluN3A located at the “B” and “D” positions adopts a “1,3,1,3” conformation and the existence of a homophilic intersubunits interface between GluN3A-NTDs. However, the subunit geometry of “1,1,3,3,” “1,1,1,3,” “1,3,3,3” cannot be excluded based only on biochemical results. Providing a high-resolution protein structure of these receptors may be the simplest approach to clarify these hypotheses.

**Figure 3.**
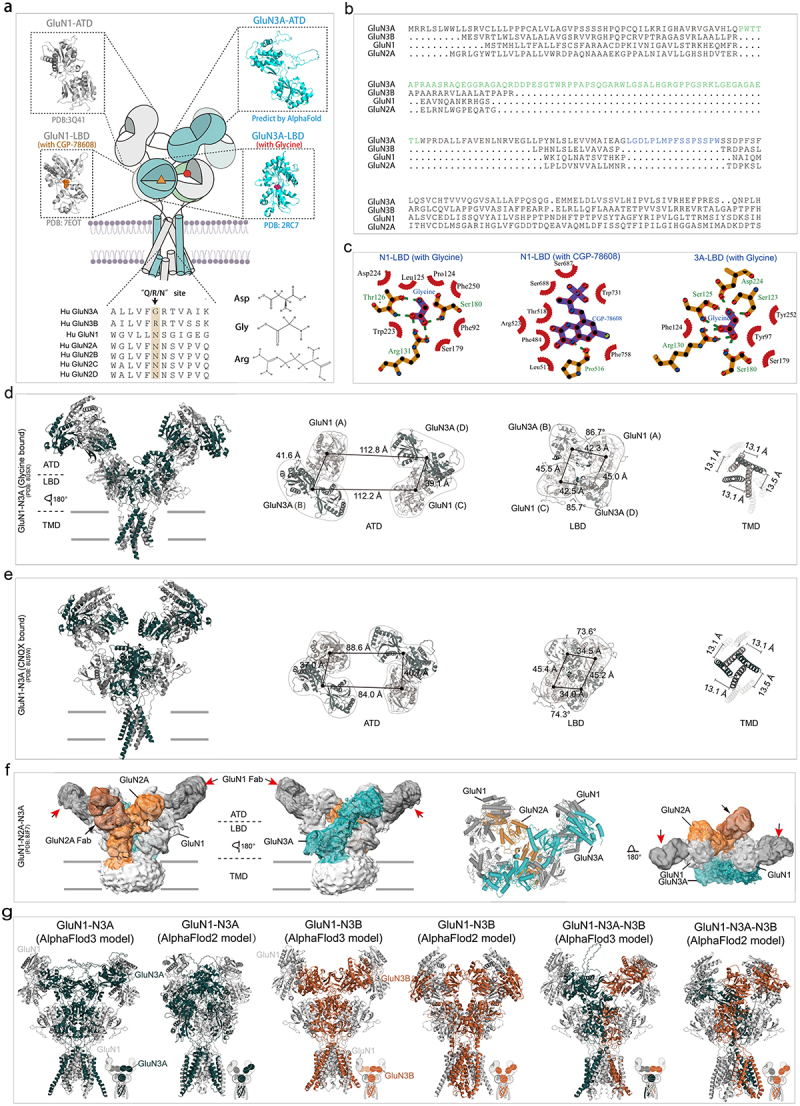
Structural basis, sequence alignment, and Ligplot^+^ analysis of GluN1-N3 NMDA receptors. a. Structural model of GluN1-GluN3. b. Particle sequence alignment of GluN1, GluN2, and GluN3 (the predicted loop was shown in green and blue, respectively). c. A schematic illustrating ligand-protein interactions of glycine/CGP-78608-GluN1-lbd and glycine-GluN3A LBD. d, e structural model of glycine bound (d) or CNQX bound GluN1-N3A(e). f. density map and structure model of GluN1-N2A-N3A. g. predicted GluN1-N3A, GluN1-N3B, and GluN1-N3A-N3B structure models. Excerpt from PDB(8JF7) (f), [25] (g). Panel (g) has been reproduced with permission from Frontiers.

The NTD structure of GluN3A is longer than that of other subunits by ~ 100 amino acids, and it may possess a distinct 3D structure. The extra ~ 100 amino acids are predicted to form a flexible linker structure by Swiss-model and AlphaFold (Figure 3(a,b)). The LBD structure of GluN3A has been elucidated in apo, agonist-bound, or antagonist-bound state by X-ray crystallography [32,33], suggesting the gating cycle of GluN3A subunit. The LBD of GluN3A shares a similar conformation with GluN1 and GluN2 subunit, with two similar but asymmetric units arranged as a head-to-tail bilobed clamshell (Venus’s flytrap)-like structure (Figure 3(a)). Upon binding to glycine, the distance of the D1-D2 lobe becomes smaller, changing the overall structure from flexible open-cleft, with a broad free energy basin, to stable close-cleft conformation, with a narrower free energy basin. This conformation switch, probably due to the glycine-binding pocket located at the inter-cleft, underlies a rich spectrum of hinge bending, rocking, twisting, and sweeping motions. However, interestingly, the rotation degree of D2 relative to D1 for GluN1 is about 25°, while in GluN3A it is only 8°, during the open-to-close cleft shift, indicating that the LBD of GluN3A maintains a relative more condensed conformation than GluN1’s in the apo-state [33]. This observation reveals the unique framework of apo to agonist binding cycle in the LBD, and it may be safe to assume that GluN3 possesses a disparate apo-open-desensitization receptor cycle. GluN3A has a “G” and GluN3B has a “R” in the “QRN” site of TMD, which is assumed to control the ion permeability of iGluRs. While other subunits of NMDARs possess an “N” in this site (Figure 3(a)). The LigPlot^+^ analysis showed that Thr126, Arg131, and Ser180 form hydrophobic interactions with glycine in the GluN1 LBD. In the GluN3A LBD, the hydrophobic interactions with glycine involve Ser123, Ser125, and Arg130. The glycine site competitive antagonist CGP-78608 can form interactions with Pro516, Ser687, Trp731, and Phe758 (Figure 3(b,c)).

The first structure of a GluN1-N3A receptor was resolved in 2024, providing both glycine-bound active and CNQX-bound inhibited states (Figure 3(d,e)) [34]. Liu et al. also predicted the theoretical apo structures of GluN1-N3A and GluN1-N3B using AlphaFold, as well as the structure of GluN1-N3A-N3B [25]. Overall, these structures, like conventional NMDARs, form a tetrameric bouquet structure arranged as GluN1-N3-N1-N3. Due to the longer NTD in GluN1-N3A, the NTD of this receptor appears highly unstable, as evidenced by the lower resolution of the NTD in GluN1-N3A cryo-EM data. This instability is supported by further findings, where GluN3A was not well-defined in the GluN1-N2A-N3A structure (PDB: 8SJF) (Figure 3(f)). As a result, in the active state, the distance between the center-of-mass (COM) of GluN1-N1 and GluN3-N3 in the NTD is about 30 Å longer than in the inhibited state. This loose arrangement of subunits in the active state is even reflected in the LBD, where the total distance between the LBDs in the active state is much longer than in the inhibitor state. Unfortunately, the TMD region remains unchanged between the inhibited and active states (Figure 3(d,e)).

However, cryo-EM structures of GluN1-N3B and GluN1-N3A-N3B have yet to be reported. The AlphaFold-predicted structures indicate a significant similarity in subunit arrangement and domain interactions between GluN1-N3A and GluN1-N3B in the apo state. Notably, AlphaFold Multimer [35] and AlphaFold 3 [36] showed considerable differences in predicting GluN1-N3 structures, and Liu et al. demonstrated the accuracy of AlphaFold multimer predictions using cysteine crosslinking experiments (Figure 3(g))

There is evidence supporting the existence of GluN3-containing tri-heteromeric NMDARs *in vitro* or *in vivo*, though the atomic structure of these receptors has not yet been determined. Lv et al. provided the structural basis for the tri-heteromeric GluN1-GluN2A-GluN2B through cryo-EM and found that the GluN2A subunit plays a dominant role in the gating cycle [37]. The first tri-heteromeric GluN1-N2-N3 receptor (PDB: 8JF7) was purified by a two-step of affinity purification and its stuctural basis was then elucidated by cryo-EM. This structure consists of two GluN1 subunits, one GluN2A subunit, and one GluN3A subunit, exhibiting characteristics of both GluN1-N2 and GluN1-N3 NMDARs. However, due to the mobility of the GluN3A subunit, the resolution of this structure is limited. The introduction of GluN3A results in asymmetry at both the NTD and LBD levels, with the GluN3A subunit adopting a more parallel orientation to the cell membrane.

## The channel biophysical properties

### The channel biophysical properties of di-heteromeric GluN3-containing NMDARs

In *in vitro* system harboring GluN1-N3A or GluN1-N3B, the application of low concentrations of glycine evokes a quick inward current response. Co-application of glutamate with glycine does not show an additive effect, while the application of glutamate alone cannot evoke measurable current [7]. The current and ion permeability of iGluRs is assumed to be controlled by the key “Q/R/N” site of the selectivity filter, a short α-helix of reentrant TM2 that bends from the inner membrane surface toward the center of the channel and followed by a stretch of amino acids that returns to the inner surface. As mentioned above, in GluN1 and GluN2 subunits, the presence of a non-charged N at the Q/R/N site endows high Ca^2+^ permeability. While in GluN3, however, the presence of a G and R instead of N at this homologous position suppresses both Ca^2+^ permeability and block by Mg^2+^ and probably polyamines (Figure 3(a)). The ions mediating GluN1-N2 currents are Ca^2+^ and Na^+^, while for GluN1-N3, the major is Na^+^, since the Ca^2+^ to Na^+^ permeabilization index (P_Ca_^2+^/P_mono_) for GluN1-N2A and GluN1-N3A is 2.4 [15] and 0.8 [19], respectively.

**Figure 4.**
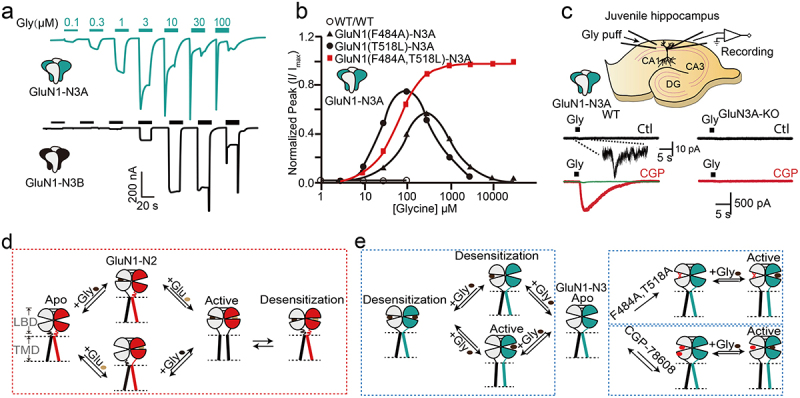
Representative channel currents of GluN1-N3 and activation model. a. The current of GluN1-N3 was measured from the Xenopus laevis oocytes. b. The concentration-response relationship of GluN1-N3A with or without F484A, T518L, or both mutants in the Xenopus laevis oocytes. c. Native GluN1-N3A recording in the juvenile hippocampus from wild-type or GluN3A-KO animal by preincubation of CGP-67808. d. The activation schematic diagram of GluN1-N2A. e. The activation schematic model of GluN1-N3A with or without the presence of double mutants or CGP-78608. Excerpt from [7] (a), [40] (b), [45] (c), and these figures have been reproduced with permission from Springer nature (a), Elsevier (b), Nature Portfolio (c).

In recombinant system the concentration of glycine for activation of GluN1-N3 is very low, with the EC_50_ (Half-maximal effective concentration) for GluN1-N3A is ~1 μM and for GluN1-N3B is ~5 μM [7] (Figure 4(a)), while the native concentration of intrinsic glycine is ~10 μM [38]. To this end, the constitutive GluN1-N3 will be in a constantly “active” (open or desensitization) state in the brain. This seems best interpreted by assuming the presence of additional unknown auxiliary proteins or unidentified mechanism modulating these types of receptors in native tissues. According to single-channel recording data, GluN1-N3B opens to two levels with chord conductances of 37 ± 0.8 pS and 12 ± 0.8 pS [7], while the chord conductance in GluN1-N2A is 75 ± 2.5 pS and 6.0 ± 2.5 pS [39].

Another notable feature of GluN1-N3A and GluN1-N3B currents is their rapid desensitization. Rapid desensitization occurs above ~3 μM and ~10 μM for GluN1-N3A and GluN1-N3B, hampering the detailed analysis of channel character at high glycine concentration, and potentially leading to the inability to detect in early studies and native tissues. Several approaches have been developed to overcome this challenge: 1) mutations on amino acids critical to glycine-binding pocket to disrupt glycine binding to GluN1 subunit. The F484A and T518L mutations of GluN1 dramatically potentiate the peak and steady-state currents while suppressing the desensitization of GluN1-N3A and GluN1-N3B receptors [40–42] (Figure 4(b)). 2) pre-incubation with the high-affinity GluN1 antagonist CGP-78608 (which can bind to GluN3A with much lower sensitivity) or MDL-29951 (Figure 4(c)) [41,43]. 3) Combination with different splice variants of GluN1 subunit. The receptor formed by GluN1-4a and GluN3A exhibits a larger current and longer desensitization time under the same glycine stimulation compared to the receptor formed by GluN1-1a and GluN3A [44]. Notably, it appears that the NTD of GluN3A contains a Zn^2+^ sensor. Studies have shown that Zn^2+^ can enhance the current of GluN1-N3A receptors and slow their desensitization [43]. Patch-clamp studies using CGP-78608 [45] isolated native GluN1-N3A currents in juvenile hippocampal slices, providing the first direct evidence of functional GluN1-N3 receptors in native tissues (Figure 4(c)). This finding helped clarify the physiological roles of these receptors, previously considered restricted to heterologous expression systems.

The steady-state current of GluN1-N3A in the presence of 1 mM glycine can be enhanced more than 1000-fold and the desensitization can be greatly reduced by preincubation with 500 nM CGP-78608 (Figure 4(c)) [45]. Based on these studies, the “apo-activate-desensitize” cycle for GluN1-N3 should be distinct from GluN1-N2 receptors. The binding of glycine to GluN1 and GluN3 results in two separate effects on the receptor function: glycine binding to high-affinity GluN3 sites activates the ion channel, while glycine binding to low-affinity GluN1 sites leads to desensitization or pre-desensitization of the channel. Hence, glycine co-binding to GluN1 and GluN3 leads to a rapid transition from the open to the desensitized state. The mechanism underlying the effects of two mutations and glycine-binding site antagonists on GluN1-N3A current likely involves preventing glycine binding to GluN1, thereby abolishing the open-to-close-cleft conformation shift, promoting pore opening, and preventing desensitization (Figure 4(d,e)).

Notably, GluN1-N3A exhibits reduced sensitivity to conventional NMDARs channel pharmacological tools, such as the open-channel blocker memantine [46] and MK801 [47,48], which maybe the simplistic explained by the distinct pore architecture of GluN1-N3 (Figure 3(a)). However, as mentioned above, blocking the binding of glycine to GluN1 abolished the rapid desensitization of GluN1-N3A and GluN1-N3B. Therefore, it is logical that 5,7-DCKA [46] and CNQX [49], two GluN1-specific glycine site antagonists, play roles in modulating GluN1-N3 currents. Nevertheless, only few allosteric molecules targeting GluN3 are available, including: TK13, TK30 [40], and EU1180–438 [50]. TK13 targets both GluN1-N3A and GluN1-N3B, while TK30 specific noncompetitive antagonized GluN3A. EU1180–438 is a noncompetitive antagonist with an activity that is independent of membrane potential. Due to the strategy of pre-incubation of CGP-78608/MDL-29951 and the 484/518 mutations in GluN1, there has been significant interest in developing and screening new GluN3-specific pharmacological tools via high-throughput calcium imaging, including both positive and negative allosteric modulators and channel blockers [51].

### The channel biophysical properties of tri-heteromeric GluN3-containing NMDARs

**Figure 5.**
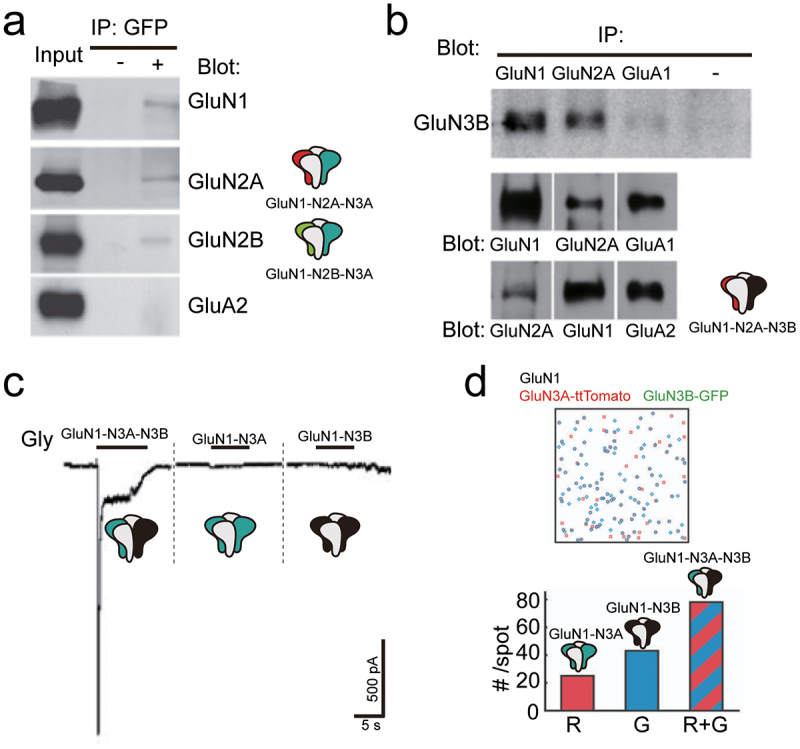
Evidence for the presence of GluN3-containing triheteromeric NMDARs. a. The GluN1, GluN2A, and GluN2B subunits were immunoprecipitated with GluN3A (fused with GFP). b. The GluN1 and GluN2A subunits can be immunoprecipitated with GluN3B. c. The current of GluN1-GluN3A, GluN1-GluN3B, and GluN1-N3A-N3B are expressed in the Xenopus laevis oocytes. d. The assembly of GluN1, N3A, and N3B subunits. Excerpt from [55] (a), [52] (b), [47] (c), [57] (d), and these figures have been reproduced with permission from Elsevier (a), Society for Neuroscience (b), ASPET (c). and National Academy of Sciences (d).

Multiple lines of evidence based on functional and morphological studies, have demonstrated the existence of GluN3-containing tri-heteromeric NMDARs (GluN1-N2-N3A or GluN1-N3A-N3B) (Figure 5(a,b)). There is evidence indicating that the GluN3A subunit cannot be incorporated into the PSD complex if lacking the GluN2 subunit in the recombinant expression system [23,52]. Using Ca^2+^ imaging or electrophysiological methods, the GluN3 subunit was found to reduce the inward current, single-channel chord conductance, and Ca^2+^ permeability of NMDARs [10,48,53]. In a study that combination Ca^2+^ imaging and cell viability measurements revealed, the incorporation of the GluN3 subunit was found to reduce cell toxicity resulting from the high concentration of NMDA incubation [54]. These results may indicate that the existence of tri-GluN1-N2-N3 NMDARs in the brain and suggest that the GluN3 likely plays negative roles in these receptors. Two main reasons may contribute to the negative roles of GluN3: Firstly, the GluN3A may reduce the expression, assembly, and membrane insertion of GluN1 and GluN2. Secondly, tri-GluN1-N2-N3 NMDARs constitute main proportion over the di-GluN1-N2 and di-GluN1-N3 in the three-subunits co-expression system. Studies supporting the first view include the following: the excitatory post-synaptic current (EPSC) in GluN3A knockout animals is larger than the wild-type littermate, and the value in wild-type animals is larger than in GluN3A over-expression animals [27,55]. Nevertheless, it remains debated whether GluN3A affects the expression of other subunits. There is evidence supporting that knockout of GluN3A reduces the expression levels of GluN1, GluN2A, GluN2B subunits [56]. However, work by Das S et al. demonstrated that GluN2A is expressed at the same level in GluN3A knockout and wild-type animals [46]. There is also evidence suggesting that GluN2 subunit facilitates the membrane insertion of GluN3B [23]. It is noteworthy that coexpression of GluN1, GluN3A, and GluN3B can significantly increase NMDAR currents and decrease the desensitization concentration of glycine [47], suggesting the existence of GluN1-N3A-N3B. Furthermore, coexpressing GluN1, GluN3A, and GluN3B results in a larger current compared to expressing GluN1 with either GluN3A or GluN3B alone (Figure 5(c)). Additionally, GluN1-N3A-N3B, with a fixed stoichiometry of two GluN1, one GluN3A, and one GluN3B, can be detached by an elegant single-molecule fluorescence colocalization method in the *Xenopus laevis* oocytes (Figure 5(d)) [57]. This type of NMDARs may have a unique gating mechanism and channel properties, seems that two GluN3 subunits have positive feedback in the gating cycle, which may differ from di-GluN1-N2, di-GluN1-N3, and tri-GluN1-N2-N3, albeit a rare report focused on these types of NMDARs. Nevertheless, it is very challenging to separate these single types of tri-NMDARs *in vivo*. Based on the above observations, it can be concluded that tri-GluN1-N2-N3 and tri-GluN1-N3A-N3B exist in the brain and possess unique biophysical properties.

## Physiological and pathological roles of GluN3-containing NMDARs

### Functional properties of GluN3A-Containing NMDARs

The GluN3A subunit has been shown to play a negative role in neuronal development as the number of dendritic spines is reduced in GluN3A-overexpressing animals compared to wild-type animals [27]. Therefore, GluN3A subunit is considered a “molecular brake” responsible for suppressing the overdevelopment of neurons [19]. This is inconsistent with the expression pattern of GluN3A subunit, which is highly expressed in the early development state [11]. In addition, an autopsy study found a lower GluN3A subunit expression in premature infants [58], supporting that the GluN3A subunit plays an important role in brain development. Functional studies have shown that GluN3A subunit plays a role in EPSCs and is therefore a vital player of synaptic plasticity [59]. Neurons from GluN3A subunit knockout mice exhibited enhanced NMDA-induced currents and EPSCs with reduced sensitivity to Mg^2+^ [56], Further studies revealed that GluN1-N3A plays key roles in regulating neuronal excitability in the medial habenula, a brain region involved in emotional control [60,61]. In contrast, GluN3A subunit overexpression in mice showed opposite effects [26]. Subsequent studies revealed that the GluN3A subunit played key roles in learning and memory [62,63], emotional adjustment [60], movement [64], and sensory control [65]. Further signaling studies have shown that GluN3A may act as regulatory target of the calcium-response factor (CaRF) and can therefore be downregulated by elevated intracellular Ca^2+^ levels [66]. Another study using multi-methodologies revealed that the existence of a GluN3A-p38MAPK-MEF2C signaling pathway, through which GluN3A modulates the expression of several genes [67]. An interesting study revealed that *GRIN3A* is a target of interferon-alpha [68], a cytokine, that plays vital role in many biological activities, such as anti-SARS-CoV2, and anti-cancer effect. Notably, one study has shown that individuals with high working memory have a higher level of *GRIN3A* [69]. Moreover, the GluN3A subunit is involved in myelination [70].

During ischemic brain stroke, distinct roles, both beneficial and detrimental, of GluN3A have been reported (Table 1). The Mg^2+^ less sensitive NMDARs current and Ca^2+^ accumulation in the central myelin led to oligodendrocyte damage, which has been mediated by GluN3A-containing NMDAR [71–73]. On the contrary, a lot of work supports the beneficial effect of GluN3A in brain diseases. In a study that knockout GluN3A reduced cell damage in the ischemia stroke in both cultured neurons and *in vivo*, while overexpression GluN3A exacerbated cell damage [74,75]. In Huntington’s disease (HD) animal models, the dysfunction of GluN3A-containing NMDARs has been considered a main cause for cell damage, while HD protein PASCIN1 signaling leads to the overexpression of GluN3A, further exacerbating cell damage. Supportive studies have shown that overexpressed GluN3A can mimic several symptoms and knockdown GluN3A attenuation behavior impairments in HD mouse model [76–79]. Yuan et al found the GluN3A-containing NMDARs with low Ca^2+^ permeability, are expressed in dopaminergic neurons and increased in the Ventral tegmental area after cocaine exposure [80]. Additionally, another study focusing on mRNA sequencing found that rs17170632 site C to A single nucleotide polymorphism is related to heroin addiction [81]. Moreover, *GRIN3A* was increased in the peripheral blood lymphocyte of game players but decreased in the hippocampus and orbitofrontal cortex of individuals with alcohol addiction [82]. These results strongly indicate that the GluN3A subunit plays an imperative role in substance addiction (Table 1).

**Table 1.** Diseases related to GluN3-containing NMDARs.

Disease	Key GluN3-containing NMDARs alternation	Roles of conventional NMDARs	Reference
Depression	GluN3 expression decreases in hippocampal	Overexpression and hyperactivity of GluN2-containing NMDARs	[83,84]
	GluN3 expressions increase in medial prefrontal cortex		
Ischemia stroke	GluN3A involves Ca^2+^ overload	Excessive GluN1-N2B activation leads to excitotoxicity	[71–75,93]
	Overexpression of GluN3A reduces cell death		
	Overexpression of GluN3A exacerbates cell damage		
	Overexpression of GluN3B is protective		
Huntington’s disease	The HD protein-PASCIN1 signaling leads to overexpression of GluN3A	Extrasynaptic GluN1-N2B activation causes mutant Huntington protein-induced cell damage	[76–79]
	Knock down or suppressing GluN3A expression attenuates disease phenotypes		
Addiction	GluN3A containing NMDARs increases in cocaine addiction	Excessive NMDARs activation in limbic regions involves reinforcement of cocaine	[80–82,99,100]
	Single nucleotide polymorphism of GluN3A relates to heroin addiction		
	GluN3A is upexpressed in game players		
	GluN3A is downexpressed in alcohol addiction		
	GluN3B is upexpressed in opiate addiction		
Autoimmune encephalitis	Existence of antibodies against GluN3B and antibody titles is positive correlation to disease seriousness	Decreasing distribution of NMDAR in cell surface impairs synaptic function	[94]
Subarachnoidhemorrhage	GluN3B is downexpressed in disease	Excessive GluN1-N2B activation leads to excitotoxicity	[97]
Schizophrenia	*GRIN3B-rs2240158* mutation correlates with the auditory mismatch negativity	Down-expression of activity of NMDARs in GABAergic neurons leads to an imbalance in neural network	[101,102]
Alzheimer’s disease	GluN3A modulates Ca^2+^ influx	Overexpression and hyperactivity	[85–87]
	GluN3A impacts Tau protein expression		

Furthermore, reduced expression of GluN3A-containing NMDARs has been shown to result in depression-like behavior [83,84]. There is evidence suggesting that GluN3A regulates neuronal calcium levels, preventing excessive neuronal activation, subsequent calcium overload, neuroinflammation, and impaired synaptic integrity/plasticity, thereby protecting against sporadic Alzheimer’s disease (AD) [85]. Moreover, evidence also supports GluN3A’s involvement in late-onset AD through modulation of Ca^2+^ influx [86], and GluN3A deficiency impacts Tau protein expression levels [87]. However, some evidence suggests that in AD animal models, there is no significant change in the expression or distribution of GluN3A in synapses [88], indicating no clear evidence for its impact on AD [89]. In addition, GluN3A expression levels in patient samples from dysembryoplastic neuroepithelial tumors were found to be significantly higher than in non-patient samples [90]. It is noteworthy that the pathological role of GluN3A is not limited to the nervous system, as it is also a biomarker in prostate cancer [89] and skin cutaneous melanoma (Table 1) [91].

### Functional properties of GluN3B-Containing NMDAR

In the physiological processes, the GluN3B subunit, in contrary to GluN3A, may act as a “molecular accelerator,” which is proved to promote development. Overexpression of the GluN3A subunit in the cultured motor neurons could increase the length and complexity of dendrite branches [22]. A sequencing study found that the GluN3B has four mRNA splices, and co-expression of these mRNA splices could result in distinct channels [53], indicating the GluN3B subunit has a fine-turning modulation property in neurons. The further functional study by gene manipulation found learning and memory deficits, depression-like behavior, and increased motivation in *Grin3b* knockout animal under new circumstances [92]. Moreover, a sequencing study targeting European-American people found the evolutionary speed of GluN3B was higher than other genes and had a frameshift mutation between exon 3 and exon 4, which led a truncated GluN3B subunit.

GluN3B may have a neuroprotective effect in several brain diseases (Table 1). In a study focusing on cerebral ischemia, the expression of GluN3B was found to increase, while GluN1, GluN2A, and GluN2B decreased, and the GluN3B can be further increased with neuroprotective progesterone treatment [93]. This upregulation suggests that GluN3B may possess a neuroactive role during the pathology of cerebral ischemia. One study on autoimmune encephalitis found that antibodies against GluN3B existed in the patients’ cerebrospinal fluid. Moreover, the patients showed more severe symptoms if the antibody titers were at a high level meaning that normal expression of GluN3B on the cell membrane is a prerequisite of normal neuronal activity [94].

A study found fetal mice that received radiation of specific wavelengths suffered from cognitive dysfunction after birth and were accompanied by increased levels of *Grin3a* and *Grin3b* mRNA [95]. However, evidence also exists demonstrating the significant reduction of GluN3B expression in disease models [96]. For example, the expression of the GluN3B, as well as GluN2A, GluN2B subunit decreased in the model of subarachnoid hemorrhage [97]. Exposure of pregnant mice to air pollution substances led to the decrease of GluN3B expression, accompanied by an increase of inflammatory reaction and cognition deficits of the next generation [98]. The GluN3B, like the GluN3A subunit, may also be involved in the pathophysiology of addiction. Evidence includes that: 1) the mRNA level of GluN3B was significantly higher in opiate addiction patients [99] and rat with morphine administration [100]. 2) C-T polymorphism of RS2240158 in *GRIN3B* of heroin addiction patients was significantly different from that of healthy controls [81]. GluN3B may also be involved in psychiatric disorders. In humans, *GRIN3B*-rs2240158 mutation was associated with the auditory mismatch negativity, which is known as the intrinsic indicator of schizophrenia [101]. This hypothesis can be further supported by the whole genome sequencing of familial psychiatric disorders, which found the frameshift mutation of *GRIN3B*-rs10666583 was significantly higher than that of the healthy controls [102]. This mutation abolished the binding of glycine to the GluN3B subunit, which in turn affected the function of the receptor, especially the permeability of the channel to Ca^2+^.

## Conclusion and Outlook

In contrast to the well-characterized structural and functional properties of conventional GluN1-N2 NMDARs, our understanding of GluN3-containing NMDARs remains limited, with only a few having been identified and studied to date. It may not be fully appropriate to apply the knowledge from GluN1-GluN2 NMDARs to the understanding of GluN3-containing NMDARs. The differences in protein sequences also suggest that the three-dimensional atomic structure and gating mechanisms of GluN3-containing NMDARs are significantly different from those of conventional NMDARs. In addition to differences in spatio-temporal distribution, cell type, and subcellular structure, these differences suggest a potentially novel function for GluN3-containing NMDARs.

Two major directions for future research may include:

- To explore the similarities and differences between GluN3-containing NMDARs and conventional NMDARs at both structural and functional levels. Recent structural biology studies of GluN3-containing dimeric and trimeric receptors, as well as AI-predicted structural models, have revealed that the arrangement of sub-domains in the GluN3A subunit, interactions with the GluN2 subunit, and the amino acids forming the ligand-binding pocket differ significantly from those of GluN1-containing receptors. These differences are reflected in functional aspects, such as the rapid activation-desensitization-inactivation cycle and significantly reduced low calcium permeability. Pathophysiologically, the abnormal expression of GluN3 has been implicated in various diseases, including Huntington’s disease, Alzheimer’s disease, ischemic stroke, depression, and other neurodegenerative diseases. However, many questions remain to be explored, such as the structural and functional basis of its activation-inactivation cycle, its role in brain development and maintenance, its involvement in other neurodevelopmental abnormalities, and its impact on other neurological diseases. There is also emerging evidence of its high expression in other areas, the physiological and pathological significance of which warrants further investigation.
- Investigating the native composition and subunit distribution of GluN3-containing NMDARs. It is crucial to examine the native complex of GluN3-containing NMDARs, as recent findings suggest significant differences in their subunit composition and the signaling pathways they mediate compared with traditional NMDA receptors. This opens exciting new avenues for understanding the biological diversity and underlying principles of these receptors.

## Data Availability

Data sharing is not applicable to this article as no new data were created or analyzed in this study.
